# The Role of Pharmacotherapy in Social Cognition, Empathy, and Serum Oxytocin Levels in Children with Attention Deficit Hyperactivity Disorder: A Case–Control Study

**DOI:** 10.3390/children12101367

**Published:** 2025-10-10

**Authors:** Hasibe Ozlem Pekmez, Ipek Suzer Gamli, Oguz Bilal Karakus

**Affiliations:** 1 Child and Adolescent Psychiatry Unit, Hatay Education and Research Hospital, 31180 Hatay, Türkiye; hasibeozlem.pekmez@saglik.gov.tr; 2 Child and Adolescent Psychiatry Unit, Istanbul Erenkoy Mental and Neurological Diseases Training and Research Hospital, 34738 İstanbul, Türkiye; 3 Child and Adolescent Psychiatry Unit, Trabzon Kanuni Training and Research Hospital, 41400 Trabzon, Türkiye; oguzbilal.karakus@saglik.gov.tr

**Keywords:** ADHD, pharmacological treatments, social cognition, oxytocin, empathy

## Abstract

**Highlights:**

**What are the main findings?**

- In children with attention-deficit/hyperactivity disorder (ADHD), regular pharmacological treatment with either methylphenidate (MPH) or atomoxetine (ATX) was associated with significant improvements in social skills, with ATX specifically linked to enhanced social cognition performance.
- Empathy (BEI scores) and serum oxytocin levels did not significantly differ across treatment and control groups.

**What are the implications of the main findings?**

- The findings suggest that pharmacotherapy may improve social cognition, though long-term effects on empathy or serum oxytocin levels remain unclear.

**Abstract:**

**Background/Objectives:** Attention-deficit/hyperactivity disorder (ADHD) is increasingly recognized for its impact on social functioning, including deficits in social cognition and empathy. Emerging neurobiological evidence highlights the potential role of oxytocin in these impairments. However, the influence of pharmacotherapy, particularly methylphenidate (MPH) and atomoxetine (ATX), on these domains remains underexplored. This study aimed to examine the effects of MPH and ATX on social cognition, empathy, and serum oxytocin levels in children with ADHD. **Methods:** This study included 152 children aged 6–12 years diagnosed solely with ADHD. The patient group consisted of 102 children, comprising *n* = 52 receiving MPH and *n* = 50 receiving ATX for at least 3 months. The control group comprised 50 newly diagnosed, untreated children. A sociodemographic form, the Social Skills Rating Scale (SRSS), the Reading the Mind in the Eyes Test (RMET), the Bryant Empathy Index (BEI), and the Swanson, Nolan, and Pelham Questionnaire (SNAP-IV) were applied. Serum oxytocin levels were measured via venous blood samples. **Results:** Medicated children exhibited significantly elevated SRSS scores, irrespective of the pharmacotherapy administered. RMET scores were significantly higher in the ATX group. No significant differences were found between the three groups in terms of empathy scores and serum oxytocin levels. A significant negative correlation was identified between ADHD symptom severity and RMET and SRSS-Total scores. Regular medication use was a significant predictor of SRSS scores, while empathy and serum oxytocin levels were nonsignificant predictors. **Conclusions**: Pharmacotherapy may enhance social cognition among children with ADHD. Longitudinal studies are warranted to assess the long-term effects of medication on social cognition and empathy.

## 1. Introduction

Attention-deficit/hyperactivity disorder (ADHD) is a neurodevelopmental disorder characterized by difficulties with attention, excessive activity, and impulsive behavior, which has a substantial impact on multiple functional domains [1]. Considering the significant adverse long-term consequences, the management of ADHD is critical and generally comprises a comprehensive, multimodal approach [1,2]. The treatment protocols include both pharmacological treatment and psychosocial interventions, such as behavioral therapy, parent training, and educational support [3]. Pharmacological treatment remains a cornerstone in the management of ADHD and primarily involves the use of stimulant medications, such as methylphenidate (MPH) and amphetamines. Additionally, non-stimulant medications, including atomoxetine (ATX), bupropion, guanfacine, and clonidine, are generally considered to have favorable efficacy and tolerability profiles [4]. Although international guidelines vary in their recommendations for the treatment of ADHD, there is consensus that first-line pharmacological treatment typically involves the use of stimulants. Among non-stimulant options, ATX is widely recognized as an effective alternative and is the most available and commonly prescribed non-stimulant agent in our country [5,6].

Beyond the core symptoms, research consistently indicates that children with ADHD frequently face difficulties in social functioning, particularly in forming and sustaining social relationships [7]. Research suggests that children with ADHD may struggle with reading social cues, processing social information accurately, and responding appropriately during social interactions [8]. In clinical contexts, social competence, along with enduring difficulties in initiating, maintaining, and navigating peer relationships, has been frequently reported [9].

It has been stated that difficulties in social relationships may be indirect consequences of primary symptoms of ADHD, such as impulsivity, emotional regulation difficulties, novelty seeking, and inattention [10,11]. Attentional difficulties may impede effective listening and sustained engagement during interpersonal interactions, thereby contributing to the deterioration of social relationships. Moreover, hyperactivity and impulsivity may lead to negative, aggressive behaviors or socially inappropriate, intrusive actions [12]. Due to impaired social functioning, the literature consistently reports that children with ADHD often struggle with forming and maintaining friendships, experience poorer friendship quality, demonstrate fewer positive social interactions, and are more likely to face peer rejection [13,14,15].

Research indicates that social behavior and interactions in children with ADHD are governed by a complex interplay of factors, notably involving disruptions in the regulation of monoamine neurotransmitters, including dopamine (DA) and norepinephrine (NE) [16,17]. Moreover, the prefrontal cortex (PFC), which is essential for regulating these neurotransmitter systems and executive functions, is closely associated with social behavior [18,19]. The functional interaction between the PFC and monoaminergic neurons releasing DA and NE plays a critical role in regulating neural circuits underlying social behavior, and disruptions in this connectivity are thought to contribute to social deficits observed in individuals with ADHD [20,21].

Furthermore, impaired interpersonal functioning observed in children with ADHD has been partly attributed to deficits in social cognition, reduced empathy, and dysregulation of oxytocin-related hormonal activity within the social behavior network. In this respect, social cognition is defined as the ability to understand others’ thoughts and intentions, enabling effective interpretation of behavior and communication in social contexts [22]. A fundamental component of social cognition, theory of mind (ToM), refers to the capacity to attribute mental states, beliefs, and intentions to oneself and others. ToM is generally considered a state-dependent cognitive function, subject to situational and contextual modulation. Deficits in ToM have been associated with impairments in the ability to understand others’ mental states, thereby affecting social interaction and communication [23,24]. Additionally, empathy is a multidimensional construct encompassing the ability to recognize, understand, and share the thoughts and emotions of others. Empathy typically refers to the capacity to share and understand another person’s emotional state and is often conceptualized as a trait-like characteristic, reflecting a relatively stable aspect of personality. It is considered a key component of social cognitive processes, with the development of social cognition believed to facilitate enhanced empathic responses [25,26].

Furthermore, oxytocin has traditionally been recognized for its role in maternal behaviors, including childbirth and mother–infant bonding; however, a growing body of evidence indicates that oxytocin may be critically involved in modulating social cognitive processes, including empathy, emotion recognition, and interpersonal understanding [27,28]. Although the existing research is limited and debated, oxytocin has been proposed as a potential biological marker of social cognition, as it increases attention to the eye region of human faces, enhances interpersonal trust, and improves the ability to interpret others’ emotions from facial cues, thereby contributing to the development of social skills and the facilitation of interpersonal relationships [28,29,30]. However, the association between oxytocin and social behavior has not yet been definitively established, as current evidence remains mixed and contradictory [31].

Given the critical role of social cognition in understanding and responding to social cues, impairments in these abilities are increasingly viewed as contributing to the development of psychopathology, including ADHD, where social difficulties are a common and clinically significant feature [32]. The literature has demonstrated significant deficits in ToM among individuals with ADHD, underscoring broader challenges in social cognition [33,34]. Children and adolescents with ADHD exhibit significant impairments in social cognition, particularly in the accurate recognition of facial expressions conveying anger and fear [35]. Furthermore, a recent narrative review highlights substantial impairments in both ToM and empathy among children diagnosed with ADHD, which negatively impact social interactions and interpersonal functioning [36].

Moreover, recent research indicates that children diagnosed with ADHD may exhibit significantly lower serum oxytocin levels compared with typically developing peers, indicating a possible role of oxytocin in social cognitive deficits [37,38,39]. However, findings regarding OT remain inconsistent, and some studies report no significant differences in oxytocin levels or effects between individuals with ADHD and neurotypical controls [40]. In another study, no statistically significant correlation has been found between the severity of ADHD symptoms and salivary OT levels, suggesting that dysregulation of the oxytocin system may represent a distinct clinical feature [41].

Considering the significance of these challenges, it has been proposed that social and interpersonal difficulties should be addressed as part of the treatment plan; however, existing evidence remains limited. It has been hypothesized that pharmacological treatment may alleviate difficulties in social interactions by modulating key neurotransmitter systems involved in attention, impulse control, and emotional regulation, thereby supporting more accurate interpretation of social cues, improved behavioral responses, and enhanced peer interactions. In this respect, a recent systematic review reported that MPH may lead to partial improvements in social impairments among children with ADHD [42]. In another study, administration of a single dose of MPH was associated with an improvement in ToM and social functioning in children with ADHD [43]. Faux pas recognition in children with ADHD may improve with stimulant administration [26]. Furthermore, children with ADHD may exhibit dysfunction within the oxytocinergic system, particularly regarding the responsiveness of oxytocin to social interactions [43]. Although research on the effects of non-stimulants on social cognition and empathy is limited, some evidence suggests that ATX may also exert a positive impact on facial and emotional recognition in children with ADHD [44].

To expand upon the existing literature, this study aimed to examine the effects of pharmacological treatment on social cognition, empathy, and serum oxytocin levels in children with ADHD. Specifically, this study aimed to determine whether medication used in ADHD treatment contributes to improvements in social cognitive functioning, as well as to investigate potential changes in serum oxytocin levels as a neurobiological correlate. The findings can inform targeted interventions for enhancing social skills in this population.

## 2. Materials and Methods

### 2.1. Participants

Our study was designed as an observational, cross-sectional, single-center clinical trial. A total of 152 children who met the inclusion criteria and presented as outpatients to the Child and Adolescent Psychiatry Clinic at Erenköy Mental and Neurological Diseases Training and Research Hospital’s Child and Adolescent Psychiatry Department during the six months following ethical approval were included in this study.

The patients were assessed by a child and adolescent psychiatrist, and diagnoses were made in accordance with the DSM-5. Patients were evaluated using the ‘Kiddie Schedule for Affective Disorders and Schizophrenia—Present and Lifetime Version, Turkish Adaptation (K-SADS-5)’ interview to identify any psychiatric comorbidities. The patient group consisted of two subgroups totaling 102 children who had been diagnosed with ADHD and were regularly being followed up at our outpatient clinic, comprising 52 patients aged 6 to 12 years who had been receiving MPH for at least 3 months, and 50 patients aged 6 to 12 years who had been prescribed ATX for at least 3 months. Patients with any comorbid conditions or those receiving other pharmacologic agents were excluded from this study. The case group was selected from those who had been receiving pharmacological treatment in compliance with the latest guidelines and were being monitored via monthly follow-up visits by a child and adolescent psychiatrist [45]. This study was designed to compare children receiving these treatments, the two most prescribed pharmacological agents for ADHD in our country, as well as in treatment-naïve children [6].

In this respect, 50 newly diagnosed children aged 6 to 12 years, with any treatment history, based on clinical interviews using the K-SADS-PL, who did not meet the diagnostic criteria for any psychiatric disorder other than ADHD, were included as the non-medicated group. Patients and their parents who volunteered to participate in this study were included, and informed consent forms were obtained. Following the approval of the Ethics Committee, participants in the medicated groups were consecutively selected from individuals who applied to the outpatient clinic and met the inclusion criteria. Afterward, the non-medicated ADHD group was formed by matching participants based on age, gender, and sociodemographic characteristics to ensure comparability and reduce potential confounding variables. All procedures performed in studies involving human participants followed the ethical standards of the institutional and/or national research committees and the 1964 Helsinki Declaration. Informed consent forms were obtained from the parents or caregivers of the children and adolescents taking part in this research. Ethical approval for this study was obtained.

### 2.2. Psychometric Evaluation and Measurements

Initially, a sociodemographic questionnaire was administered to gather details, including each participant’s age, birth, and developmental history, medical and educational background, social relationships, family environment, and any notable adverse experiences. Participants were then interviewed by a child and adolescent psychiatrist. The following measurements were applied to children and their parents.

#### 2.2.1. Kiddie Schedule for Affective Disorders and Schizophrenia for School-Aged Children–Present and Lifetime (K-SADS-PL)

The K-SADS-PL, initially developed by Kaufman et al. [46] a clinician-administered semi-structured interview designed to identify both current and past psychopathologies based on the Diagnostic and Statistical Manual of Mental Disorders (DSM) and its text revisions (DSM-TR). The K-SADS-PL has been demonstrated to be a valid and reliable tool for assessing psychiatric disorders in children and adolescents. Following the publication of the DSM-5, the interview protocol was revised to ensure consistency with the updated diagnostic criteria [46]. The Turkish adaptation of the instrument was validated and its reliability confirmed by Ünal et al. [47].

#### 2.2.2. Social Skills Rating Scale (SSRS)

The Social Skills Rating System, developed by Akçamete et al. [48], is a standardized assessment tool designed to evaluate the core social skills in children aged 7 to 12 years. It consists of 69 items and 12 subscales and is administered using a 5-point Likert scale, with higher scores indicating stronger social skills. The scale has been validated as an appropriate instrument for assessing social skills in Turkish children [48]. The scale is completed by the clinician via a structured interview with the parents.

#### 2.2.3. Reading the Mind in the Eyes Test–Revised Child Version (RMET)

The child version of the Reading the Mind in the Eyes Test, adapted from the original adult version developed by Baron-Cohen et al. in 1997 and validated in 2001 [49], is designed to assess advanced theory of mind, social cognition, and the ability to recognize emotions. The Turkish version was validated by Girli in 2014 [50]. The test, administered by a clinician, consists of 28 items, each presenting a pair of eyes along with four emotional descriptors, only one of which is correct. Participants receive 1 point for each correct response, with a total possible score of 28. Lower scores indicate impairments in social cognitive functioning.

#### 2.2.4. Bryant Empathy Index (BEI)

The Bryant Empathy Index (BEI) for children and adolescents is a 22-item self-report questionnaire developed by Bryant [51] to measure empathy levels in children and adolescents. It is a widely used and reliable tool for assessing empathy in this population. The Turkish adaptation and validation study was conducted by Yüksel et al. [52] Factor analysis in the validity study resulted in the removal of 2 items, producing a 20-item version.

#### 2.2.5. The Swanson, Nolan, and Pelham Questionnaire (SNAP-IV) Scale

The Swanson, Nolan, and Pelham Questionnaire (SNAP-IV) is an 18-item behavioral rating scale designed to assess core symptoms of ADHD. It consists of 9 items evaluating inattention and 9 items evaluating hyperactivity/impulsivity, each rated on a 4-point Likert scale. Originally developed in 1981, the scale was subsequently revised to align with the diagnostic criteria outlined in the DSM-IV. The SNAP-IV is widely employed in both clinical practice and research contexts as a reliable and validated tool for the screening and assessment of ADHD symptoms [53,54].

#### 2.2.6. Sample Collection

Oxytocin levels are commonly measured using biological samples such as saliva, plasma, serum, or cerebrospinal fluid, each offering distinct advantages and limitations in reflecting peripheral or central oxytocin activity [55]. Although saliva sampling is often suggested for its non-invasive and practical advantages in clinical settings, studies have shown high intra-individual variability across multiple samples collected at similar time points, raising concerns about the biological validity [56]. In our study, serum was preferred for oxytocin measurement due to its practical advantages, wide clinical availability, ease of collection, storage, and processing. Although serum processing is standardized, the coagulation process may cause nonspecific binding or degradation of peptides such as oxytocin, potentially affecting accuracy. This risk can be minimized by standardizing clotting times, processing samples rapidly, and storing them promptly at −80 °C.

Venous blood samples (10 mL) were collected from both the case and control groups between 08:00 and 11:00 in a fasting state and before medication administration. The timing of sample collection was standardized to reduce the potential impact of contextual confounding factors. Participants were reinformed of the procedural details the day before and were required to fast and be free from medication for a specified duration, and social stimulation was minimized before the sample collection. The samples were collected into red- or yellow-capped biochemistry tubes. Red- and yellow-capped tubes are commonly used for serum collection in clinical and research settings. Red-capped tubes contain no additives or clot activators and are used when pure serum is required. In contrast, yellow-capped tubes typically contain a clot activator and a gel separator, which facilitates faster clotting and cleaner separation of serum during centrifugation. While both are suitable for serum analysis, the choice between them may influence peptide stability and recovery, particularly for analytes such as oxytocin [57].

After a 20-min resting period, the samples were centrifuged at 1000 g for 15 min. The separated serum samples were stored at −80 °C until analysis. Samples stored in Eppendorf tubes to measure serum oxytocin levels were transported to the Pharmasina Biochemistry Laboratory, using cold chain transfer equipment, per procedural standards. Serum oxytocin levels were quantified using the ELABSCIENCE E-EL-0029 OT (oxytocin) ELISA kit at the same laboratory (measurement range: 15.63–1000 pg/mL; sensitivity: 9.38 pg/mL; reproducibility: coefficient of variation < 10%). The standards, samples, and alkaline phosphatase-conjugated oxytocin were pipetted into wells coated with oxytocin antibodies. During incubation, the alkaline phosphatase enzyme catalyzed the substrate, producing a colored product. At the end of incubation, the color development was stopped by adding a stop solution, and the absorbance was measured spectrophotometrically at 450 ± 2 nm. The optical density values obtained were converted to serum concentration values using a formula derived from linear regression analysis. Immediately after the measurements, the samples were destroyed in accordance with the sample disposal procedure under the research team’s supervision.

#### 2.2.7. Statistical Analysis

Data were analyzed using IBM Statistical Package for the Social Sciences (SPSS) Statistics, version 21.0. The dataset was examined for missing values and outliers before the analysis. Categorical variables are presented as frequencies and percentages, while continuous variables are reported as means and standard deviations. Normality was assessed using skewness and kurtosis values. Non-normally distributed data are presented as medians and interquartile ranges (Q1–Q3). Chi-square tests were used for categorical variable comparisons. For two independent groups, independent-samples *t*-tests were applied when parametric assumptions were met; otherwise, the Mann–Whitney U test was used. For comparisons between multiple groups, one-way ANOVA or Kruskal–Wallis tests were conducted accordingly. When a significant omnibus effect was detected, post hoc analyses were performed. Since the assumption of homogeneity of variances was not met, the Games–Howell post hoc test was applied to control for multiple comparisons. (If the homogeneity assumption had been satisfied, Tukey’s HSD or Bonferroni tests would have been considered.) Pearson or Spearman correlation analyses were used depending on the normality assumptions. Univariate and multivariate linear regression analyses were conducted to determine predictors of continuous variables. In all analyses, a *p*-value of <0.05 was accepted as the level of statistical significance.

## 3. Results

A total of 152 participants diagnosed with ADHD were included in this study. Among them, 50 were not receiving any pharmacological treatment (the non-medicated group), while 102 participants were undergoing medication treatment for ADHD. Of those in the medicated group, *n* = 52 were receiving MPH, and *n* = 50 were being treated with ATX.

The mean age in the non-medicated group was 9.53 ± 1.80 years, compared with 9.82 ± 1.61 years in the medicated group (*p* = 0.314). The male-to-female ratio was 28:22 in the non-medicated group and 66:36 in the medicated group, indicating no statistically significant differences between the groups in terms of age or gender distribution (*p* = 0.299). When ADHD subtypes were taken into consideration, there were *n* = 3 diagnosed with ADHD-Inattention (ADHD-IA) subtype, *n* = 49 ADHD-Combined (ADHD-CT) in the MPH group, *n* = 8 ADHD-IA and *n* = 42 ADHD-CT in the ATX group, and *n* = 10 ADHD-IA and *n* = 40 ADHD-CT in the non-medicated group.

The data comparing the subscale and total scores of the Social Skills Rating System (SSRS) between these groups are presented in Table 1.

Accordingly, all subscale and total scores of the SSRS were significantly higher in the medicated group than in the control group. No significant difference was found between the MPH and ATX groups in the SRSS scores for the advanced conversation, relationship maintenance skills, self-control skills, coping with aggressive behaviors, accepting consequences, giving instructions, and cognitive skills subscales. The ATX group scored significantly higher in the initiating relationships subscale than the two other groups, while the basic social skills, basic conversation skills, group work skills, and emotional skills subscale scores were significantly higher in the MPH group.

**Figure 1:**
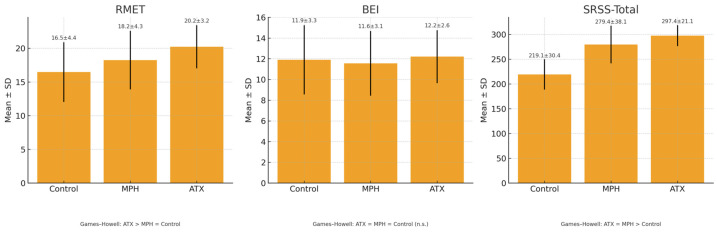
Comparison of RMET, BEI, and SRSS-Total scores in control, MPH, and ATX groups.

Data comparing the scores of the RMET and BEI between the control, MPH, and ATX groups are presented in Table 2 and Figure 1.

Accordingly, the RMET scores were significantly higher in the ATX group compared with the other two groups, while no significant difference in RMET scores was observed between the MPH and the control groups. No significant differences were found between the three groups in terms of empathy scale scores (*p* > 0.05). The control group had higher total scores on the SRSS compared with the two medicated groups; however, no significant difference was found between the MPH and ATX groups in terms of SRSS total scores (*p* > 0.05).

The serum oxytocin levels of the control, MPH, and ATX groups are given in Table 3.

Accordingly, no significant difference was found in blood oxytocin levels between the three groups (*p* > 0.05).

Table 4 presents the correlation coefficients among the continuous variables, including SNAP-IV, the scores for the RMET and BEI, and the total score of the SRSS.

Significant negative correlations were identified between SNAP-IV scores and both RMET (*r* = −0.174; *p* < 0.05) and SRSS-Total scores (*r* = −0.527; *p* < 0.001). Furthermore, a robust positive correlation was observed between RMET and SRSS-Total scores (*r* = 0.593; *p* < 0.001). No statistically significant associations were found between oxytocin levels and the other measured variables (*p* > 0.05).

Table 5 presents the results of both univariate and multivariate regression analyses, examining the predictors of social skills (SSRS-Total score).

**Figure 2:**
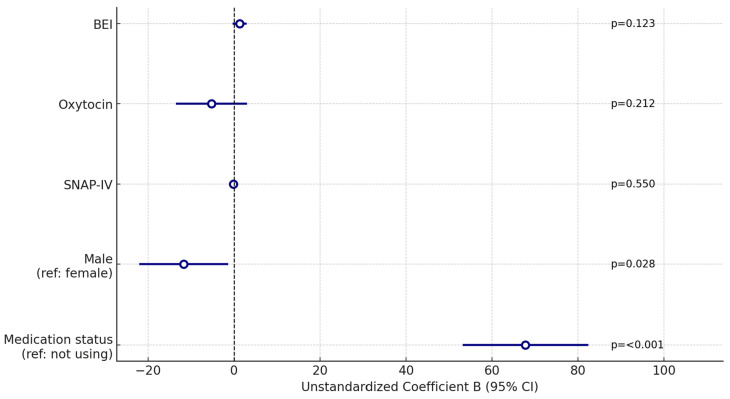
Forest plot of multivariate regression results with SSRS-Total score as the dependent variable.

The independent variables included in the model are gender (reference: female), ADHD symptom severity (SNAP-IV), empathy scores (BEI), and oxytocin levels (log-transformed). The results of the multivariate regression analysis are also illustrated in Figure 2.

In the univariate analysis, the use of medication was a significant positive predictor for SRSS-Total score (β = 0.720; *t* = 12.691; *p* < 0.001; 95% CI: [58.346, 79.865]). Gender was not a significant predictor in the univariate model (β = −0.081; *t* = −1.001; *p* = 0.318). Higher scores on the SNAP-IV (Inattention + Hyperactivity/Impulsivity) scale were significantly mediated by a decrease in SRSS-Total scores (β = −0.527; *t* = −7.592; *p* < 0.001), while empathy and serum oxytocin levels were not significant predictors.

In the multivariate model, medication status remained a significant positive predictor (β = 0.706; *t* = 7.386; *p* < 0.001; 95% CI: [53.175–82.372]). Male gender emerged as a significant negative predictor in the multivariate analysis (β = −0.126; *t* = −2.227; *p* = 0.028; 95% CI: [−22.095–1.316]). SNAP-IV scores, oxytocin, and BEI scores were not significant predictors in the multivariate model (*p* > 0.05).

## 4. Discussion

The literature consistently indicates that deficits in social cognition, empathy skills, and prosocial behaviors hinder the ability of children with ADHD to establish and sustain stable peer relationships. A negative correlation between ADHD severity and social cognition and social skills is supported by the literature, as individuals with more severe ADHD symptoms often struggle to understand social cues and may act impulsively in social interactions, leading to poorer overall social functioning. Therefore, the early identification and effective management of social impairments in this population are essential in mitigating potential challenges, including difficulties in forming and maintaining interpersonal relationships and misunderstandings in social interactions [58,59]. In the literature, it has been suggested that pharmacological treatment may alleviate social interaction difficulties by enhancing attention and impulse control, thereby enhancing the accurate perception of social cues and facilitating more appropriate responses in social situations [42]. Given that ADHD neuropharmacology primarily targets monoamine neurotransmitters, alterations involving the associated neural circuits are crucial for improving social functioning. Among approved agents, MPH primarily acts by blocking the reuptake of DA and NE, while ATX selectively inhibits NE reuptake, which are thought to be involved in facilitating social cognition, potentially via the modulation of frontostriatal and prefrontal circuits.

Supporting this, 24-week treatment with either MPH or ATX has been associated with an improvement in peer relationships alongside emotional and behavioral problems in children with ADHD [60]. This study’s findings suggest that regular pharmacological treatment and a reduction in ADHD symptom severity are significantly associated with improvements in core social skills, including communication, relationship initiation and maintenance, collaborative group work, and coping with negative social behaviors. These results are consistent with the previous literature highlighting the positive impact of symptom management on social functioning in children with ADHD. These improvements may be attributed to enhancements in executive functioning, inhibitory control, and emotional regulation, which, in turn, promote better situational awareness, reduced impulsivity, and more appropriate behavioral responses during social interactions. Pharmacological intervention may also help children perceive social cues more accurately, sustain reciprocal communication, and engage effectively in peer relationships [42,61]. Regarding social skills, as performance and fluency deficits are frequently observed in these children, treatment may primarily address performance-related impairments, contributing to a more competent and socially appropriate presentation, as supported by the findings of our study and prior research [62].

In this context, recent studies have increasingly focused on how pharmacological interventions influence social cognition in children with ADHD. A recent systematic review evaluating the impact of short-term MPH treatment on social cognition reported enhancement in ToM abilities, while findings regarding its influence on the recognition of nonverbal emotional expressions remain inconclusive [42]. However, the existing literature remains limited regarding the impact of different ADHD treatment protocols. Our study suggests that ATX may have a more favorable effect on social cognition compared with MPH. In the literature, though scarce, current evidence shows no significant difference in emotional expression between MPH and ATX treatments; however, greater improvement was observed in patients who switched from MPH to ATX [63]. It has been proposed that ATX may specifically enhance PFC function, which is critical for social cognition and emotional processing, thereby improving the ability to infer emotions from subtle facial cues, such as those in the eye region [64]. Additionally, ATX’s modulation of NE pathways might influence attentional control and emotional regulation differently than stimulants, leading to improved performance on tasks such as the RMET. Another possible explanation is that the difference in the action mechanism and the continuous symptom control provided by ATX may be associated with a more stable social engagement across different settings, compared with children receiving MPH. Moreover, the differential impact of ATX on oxytocinergic systems involved in social cognition could also play a role, although this remains to be elucidated. Although larger studies with extended follow-up are needed to clarify the effects of both treatments on these parameters, it may be beneficial to assess social skills—or at least consider potential difficulties in social functioning—as part of the clinical evaluation while initiating ATX.

In the literature, in the context of social cognition, a significant increase in empathy scores has been reported in children with ADHD receiving MPH [65]. In another study, treatment improved both affective and cognitive empathy scores in children with ADHD, and improvement in inattention scores during MPH significantly predicted enhancement in affective empathy [66]. In contrast, another study found no significant change in empathy skills following a 12-week MPH treatment [67]. Consistent with these mixed findings, our results did not reveal a significant direct association between pharmacological treatment and empathy skills; however, empathy levels were a significant predictor of improvements in social skill scores. Therefore, individuals with higher levels of empathy may experience greater improvements in social skills within treatment and should be considered during the management.

Moreover, emerging evidence suggests that both noradrenergic and dopaminergic systems may also interact with the oxytocinergic system, though through potentially distinct mechanisms. In the context of social cognition, emerging research underscores a complex interplay between social cognition, empathy, and oxytocin, suggesting that oxytocin may play a critical role in modulating empathic responses and social cognitive processes [28]. Lower serum oxytocin levels have been observed in children with ADHD, and serum oxytocin levels were found to be significantly negatively correlated with aggression and positively correlated with empathy scores in this population, suggesting a potential role in social–emotional functioning [44]. In another study, children with ADHD exhibited significantly lower serum oxytocin levels compared with healthy controls, and serum oxytocin levels were significantly lower in drug-naïve ADHD patients than in those receiving pharmacological treatment, suggesting a potential role for oxytocin in the pathophysiology of ADHD [39]. In contrast, recent research has shown that children with ADHD exhibit elevated salivary oxytocin levels relative to healthy controls; however, these increased levels were not associated with deficits in empathy or executive functioning [68]. In another study, no significant differences were found in baseline salivary oxytocin levels between children with ADHD and healthy controls. However, oxytocin levels were significantly lower in children with ADHD following parent–child interactions, and this difference was attenuated with MPH treatment, suggesting that oxytocin may serve as a mediator of social deficits in children with ADHD [43]. Our findings suggest that social cognition scores, empathy skills, and pharmacological treatment in children with ADHD may not have a statistically significant effect on serum oxytocin levels. Given the observed results indicating no significant changes in oxytocin concentrations, social behavioral impairments in children with ADHD may be modulated by other neurobiological mechanisms. Alterations in monoamine neurotransmitter systems, along with genetic predispositions and environmental factors that interact with these neurochemical systems, play a critical role in shaping social behavioral outcomes. Therefore, the multifactorial nature of social dysfunction in ADHD suggests that treatment approaches targeting multiple neurotransmitter systems and considering broader biopsychosocial contexts may be necessary.

## 5. Conclusions

In conclusion, social impairment is a key concern in children with ADHD and should be routinely assessed during evaluations. While pharmacological treatment may alleviate the core symptoms of ADHD, monitoring should extend beyond these symptoms to include social skill development.

Additionally, psychosocial interventions, such as social skills training and family–school collaboration, are essential for addressing these impairments and improving social cognition, empathy, and overall social functioning in this population. Although oxytocin plays a significant role in social interactions, research exploring its relationship with pharmacological treatment remains limited. Our study serves as a preliminary step for future research with larger sample sizes and longer follow-up periods, aiming to further investigate the impact of ADHD treatment on serum oxytocin levels and to explore the potential of serum oxytocin as a biomarker in the diagnosis or treatment of ADHD.

Our study had several limitations. The cross-sectional design and data collection from a single center may restrict the generalizability of the findings to broader populations. Although biological markers were included, oxytocin levels were measured at only a single time point, precluding analysis of dynamic changes or reactivity to social context. Measuring oxytocin in serum may be a limitation, as peripheral oxytocin concentrations may not accurately reflect central nervous system activity. Additionally, other outcome measures relied on self-report, which introduces the potential for response bias, as participants may overestimate or underestimate their behaviors. Additionally, this study focused exclusively on children, excluding adolescents and adults with ADHD, which limits its applicability across different age groups. Furthermore, only children treated with MPH or ATX were included, excluding those on other approved pharmacological treatments or combination therapies, which may further constrain generalizability. Since our study consisted of patients who were already initiated on MPH or ATX and were in the follow-up phase, the lack of access to medical data regarding initial symptom severity or treatment protocols can be considered a limitation. Hence, the cross-sectional nature of this study limits our ability to determine whether the observed improvements in social cognition are permanent. Furthermore, although this study has several notable strengths, we cannot entirely rule out confounding factors associated with the self-selection of participants into the pharmacological versus non-pharmacological treatment groups. Longitudinal research is necessary to evaluate the potential long-term or neuroplastic effects of prolonged medical treatment on social–cognitive functioning in children with ADHD.

## Figures and Tables

**Table 1:** Comparison results of SRSS subscale scores for the control, MPH, and ATX groups.

	Non-Medicated Group (*n* = 50), Mean ± SD	MPH (*n* = 52), Mean ± SD	ATX (*n* = 50) Mean ± SD	F *	Partial Eta Squared	*p*	Post Hoc (Games–Howell)
Basic Social Skills	38.70 ± 7.33	54.67 ± 7.01	57.82 ± 4.36	126.17	0.635	<0.001	ATX > MPH > Control
Basic Communication Skills	11.98 ± 3.02	15.31 ± 3.32	17.98 ± 1.58	80.55	0.444	<0.001	ATX > MPH > Control
Advanced Communication Skills	12.94 ± 2.82	19.02 ± 4.54	20.48 ± 3.50	79.50	0.440	<0.001	ATX = MPH > Control
Initiating Relationships	21.06 ± 3.78	22.31 ± 3.00	23.18 ± 2.40	5.70	0.073	0.005	ATX > Control, MPH = ATX, MPH = Control
Maintaining Relationships	22.98 ± 3.79	26.02 ± 4.37	27.34 ± 3.06	20.10	0.190	<0.001	ATX = MPH > Control
Working in Groups	22.68 ± 7.08	28.83 ± 5.97	31.38 ± 4.62	26.29	0.273	<0.001	ATX > MPH > Control
Emotional Skills	19.44 ± 6.38	23.50 ± 5.36	25.84 ± 3.32	20.31	0.208	<0.001	ATX > MPH > Control
Self-Control Skills	17.70 ± 5.53	22.96 ± 5.26	24.26 ± 4.39	22.44	0.240	<0.001	ATX = MPH > Control
Coping with Aggressive Behaviors	13.70 ± 3.56	15.48 ± 3.35	15.92 ± 2.15	7.13	0.089	0.001	ATX = MPH > Control
Accepting Consequences	7.60 ± 2.89	10.98 ± 3.22	11.38 ± 2.51	26.95	0.258	<0.001	ATX = MPH > Control
Giving Instructions	12.76 ± 3.18	17.23 ± 2.26	17.74 ± 1.83	47.58	0.451	<0.001	ATX = MPH > Control
Cognitive Skills	17.58 ± 4.73	23.40 ± 5.36	24.04 ± 3.85	30.36	0.279	<0.001	ATX = MPH > Control

MPH: Methylphenidate, ATX: Atomoxetine, SD: Standard Deviation, F: One-Way ANOVA. * Welch test results were used due to the violation of homogeneity of variances.

**Table 2:** Comparison results of RMET and BEI scores for the control, MPH, and ATX groups.

	Control (*n* = 50), Mean ± SD	MPH (*n* = 52), Mean ± SD	ATX (*n* = 50) Mean ± SD	F *	Partial Eta Squared	*p*	Post Hoc (Games–Howell)
RMET	16.46 ± 4.43	18.23 ± 4.35	20.22 ± 3.20	12.24	0.127	<0.001	ATX > MPH = Control
BEI	11.90 ± 3.34	11.56 ± 3.12	12.20 ± 2.56	0.65	0.008	0.525	ATX = MPH = Control
SRSS-Total	219.12 ± 30.40	279.44 ± 38.07	297.36 ± 21.13	111.72	0.544	<0.001	ATX = MPH > Control

RMET: Reading the Mind in the Eyes Test, BEI: Bryant Empathy Index, SRSS: Social Skills Rating Scale, MPH: Methylphenidate, ATX: Atomoxetine, SD: Standard Deviation, F: One-Way ANOVA. * Welch test results were used due to the lack of homogeneity of variances.

**Table 3:** Serum oxytocin levels of the control (MED-), MPH, and ATX groups.

	Control (*n* = 50), Med (IQR)	MPH (*n* = 52), Med (IQR)	ATX (*n* = 50), Med (IQR)	H	Epsilon Squared	*p*
Oxytocin	50.65 (39.27–68.91)	57.55 (43.23–87.04)	46.64 (41.36–75.75)	2.94	0.01	0.230

MPH: Methylphenidate, ATX: Atomoxetine. H: Kruskal–Wallis H test; Med: median; IQR: interquartile range (reported as the 1st and 3rd quartiles).

**Table 4:** Correlations among continuous variables (*n* = 152).

Variables ^a^	RMET	SRSS-Total Score	BEI	Oxytocin
SNAP-IV	−0.174 *	−0.527 **	−0.005	−0.056
RMET		0.593 **	0.002	−0.049
SRSS-Total Score			0.094	0.042
BEI				−0.011

SNAP-IV: The Swanson, Nolan, and Pelham Questionnaire Scale, RMET: Reading the Mind in the Eyes Test, BEI: Bryant Empathy Index, SRSS: Social Skills Rating Scale, ^a^ Pearson Correlation Analysis. Note: Correlation coefficients (r) are presented in the table. * *p* < 0.05, ** *p* < 0.001.

**Table 5:** Results of the Linear Regression Model.

	Univariate Regression Analysis				Multivariate Regression Analysis			
Variables	β	t	*p*	95% CI	β	t	*p*	95% CI
Medication status (reference group: not using medication)	0.720	12.691	<0.001	58.346–79.865	0.706	7.386	<0.001	53.175–82.372
Male (reference: female)	−0.081	−1.001	0.318	−22.502–7.370	−0.126	−2.227	0.028	−22.095–−1.316
SNAP-IV	−0.527	−7.592	<0.001	−2.554–−1.499	−0.046	−0.599	0.550	−0.757–0.405
Oxytocin	<0.001	−0.003	0.998	−12.132–12.093	−0.070	−1.253	0.212	−13.516–3.026
BEI	0.102	1.261	0.209	−0.872–3.949	0.087	1.553	0.123	−0.358–2.980

Dependent Variable: SSRS-Total Score, Multivariate Analysis: F_(5,151)_ = 35.852, *p* < 0.001, Adjusted R^2^ = 0.536.

## Data Availability

The data that support the findings of this study are available from the corresponding author, [Ipek Suzer Gamli], upon reasonable request.

## Abbreviations

The following abbreviations are used in this manuscript:

ADHD	Attention-Deficit/Hyperactivity Disorder
ADHD-IA	Attention-Deficit/Hyperactivity Disorder, Inattentive Subtype
ADHD-CT	Attention-Deficit/Hyperactivity Disorder, Combined Subtype
ATX	Atomoxetine
BEI	Bryant Empathy Index
DA	Dopamine
DSM	Diagnostic and Statistical Manual of Mental Disorders
K-SADS-5-PL	Kiddie Schedule for Affective Disorders and Schizophrenia—Present and Lifetime Version
MPH	Methylphenidate
NE	Norepinephrine
PFC	Prefrontal cortex
RMET	Reading the Mind in the Eyes Test—Revised Child Version
SNAP-IV	The Swanson, Nolan, and Pelham Questionnaire
SRSS	Social Skills Rating Scale
ToM	Theory of Mind

## References

[1] American Psychiatric Association (2022). Diagnostic and Statistical Manual of Mental Disorders.

[2] Barkley R.A. (2015). Attention-Deficit Hyperactivity Disorder: A Handbook for Diagnosis and Treatment.

[3] Drechsler R., Brem S., Brandeis D., Grünblatt E., Berger G., Walitza S. (2020). ADHD: Current Concepts and Treatments in Children and Adolescents. Neuropediatrics.

[4] Cortese S., Adamo N., Del Giovane C., Mohr-Jensen C., Hayes A.J., Carucci S., Atkinson L.Z., Tessari L., Banaschewski T., Coghill D. (2018). Comparative Efficacy and Tolerability of Medications for Attention-Deficit Hyperactivity Disorder in Children, Adolescents, and Adults: A Systematic Review and Network Meta-Analysis. Lancet Psychiatry.

[5] Cortese S. (2020). Pharmacologic Treatment of Attention Deficit–Hyperactivity Disorder. N. Engl. J. Med..

[6] Oner O., Turkcapar H., Isli F., Karadag H., Akbulat A., Basci A.B., Aksoy M., Seckin C., Alkan A. (2016). Attention Deficit Hyperactivity Disorder Treatment Practice in Turkey. Klin. Psikofarmakol. Bülteni-Bull. Clin. Psychopharmacol..

[7] Nijmeijer J.S., Minderaa R.B., Buitelaar J.K., Mulligan A., Hartman C.A., Hoekstra P.J. (2008). Attention-Deficit/Hyperactivity Disorder and Social Dysfunctioning. Clin. Psychol. Rev..

[8] Mikami A.Y., Normand S. (2015). The Importance of Social Contextual Factors in Peer Relationships of Children with ADHD. Curr. Dev. Disord. Rep..

[9] Zöggeler-Burkhardt L., Embacher E.-M., Smidt W. (2023). Social Relationships, Interactions and Learning in Early Childhood—Theoretical Approaches, Empirical Findings and Challenges. Early Child Dev. Care.

[10] Humphreys K.L., Galán C.A., Tottenham N., Lee S.S. (2016). Impaired Social Decision-Making Mediates the Association Between ADHD and Social Problems. J. Abnorm. Child Psychol..

[11] Sacchetti G.M., Lefler E.K. (2017). ADHD Symptomology and Social Functioning in College Students. J. Atten. Disord..

[12] Gardner D.M., Gerdes A.C. (2015). A Review of Peer Relationships and Friendships in Youth with ADHD. J. Atten. Disord..

[13] Morris S., Sheen J., Ling M., Foley D., Sciberras E. (2021). Interventions for Adolescents with ADHD to Improve Peer Social Functioning: A Systematic Review and Meta-Analysis. J. Atten. Disord..

[14] Ros R., Graziano P.A. (2018). Social Functioning in Children with or At Risk for Attention Deficit/Hyperactivity Disorder: A Meta-Analytic Review. J. Clin. Child Adolesc. Psychol..

[15] Spender K., Chen Y.-W.R., Wilkes-Gillan S., Parsons L., Cantrill A., Simon M., Garcia A., Cordier R. (2023). The Friendships of Children and Youth with Attention-Deficit Hyperactivity Disorder: A Systematic Review. PLoS ONE.

[16] Suri D., Teixeira C.M., Cagliostro M.K.C., Mahadevia D., Ansorge M.S. (2015). Monoamine-Sensitive Developmental Periods Impacting Adult Emotional and Cognitive Behaviors. Neuropsychopharmacology.

[17] Sharp J.L., Smith M.A. (2022). The Effects of Drugs on Behavior Maintained by Social Contact: Role of Monoamines in Social Reinforcement. Front. Behav. Neurosci..

[18] Li Y., Ma S., Zhang X., Gao L. (2024). ASD and ADHD: Divergent Activating Patterns of Prefrontal Cortex in Executive Function Tasks?. J. Psychiatr. Res..

[19] Zamorano F., Kausel L., Albornoz C., Lavin C., Figueroa-Vargas A., Stecher X., Aragón-Caqueo D., Carrasco X., Aboitiz F., Billeke P. (2020). Lateral Prefrontal Theta Oscillations Reflect Proactive Cognitive Control Impairment in Males with Attention Deficit Hyperactivity Disorder. Front. Syst. Neurosci..

[20] Xing B., Li Y.-C., Gao W.-J. (2016). Norepinephrine versus Dopamine and Their Interaction in Modulating Synaptic Function in the Prefrontal Cortex. Brain Res..

[21] Brennan A.R., Arnsten A.F.T. (2008). Neuronal Mechanisms Underlying Attention Deficit Hyperactivity Disorder: The Influence of Arousal on Prefrontal Cortical Function. Ann. N. Y. Acad. Sci..

[22] Grady C.L., Keightley M.L. (2002). Studies of Altered Social Cognition in Neuropsychiatric Disorders Using Functional Neuroimaging. Can. J. Psychiatry.

[23] Baron-Cohen S. (2001). Theory of Mind in Normal Development and Autism. Prisme.

[24] Uekermann J., Kraemer M., Abdel-Hamid M., Schimmelmann B.G., Hebebrand J., Daum I., Wiltfang J., Kis B. (2010). Social Cognition in Attention-Deficit Hyperactivity Disorder (ADHD). Neurosci. Biobehav. Rev..

[25] Dvash J., Shamay-Tsoory S.G. (2014). Theory of Mind and Empathy as Multidimensional Constructs: Neurological Foundations. Top. Lang. Disord..

[26] Maoz H., Gvirts H.Z., Sheffer M., Bloch Y. (2019). Theory of Mind and Empathy in Children with ADHD. J. Atten. Disord..

[27] Walter M.H., Abele H., Plappert C.F. (2021). The Role of Oxytocin and the Effect of Stress During Childbirth: Neurobiological Basics and Implications for Mother and Child. Front. Endocrinol..

[28] Ebert A., Brüne M., Hurlemann R., Grinevich V. (2017). Oxytocin and Social Cognition. Behavioral Pharmacology of Neuropeptides: Oxytocin.

[29] Erdozain A.M., Peñagarikano O. (2020). Oxytocin as Treatment for Social Cognition, Not There Yet. Front. Psychiatry.

[30] Ross H.E., Young L.J. (2009). Oxytocin and the Neural Mechanisms Regulating Social Cognition and Affiliative Behavior. Front. Neuroendocrinol..

[31] Burenkova O.V., Dolgorukova T.A., An I., Kustova T.A., Podturkin A.A., Shurdova E.M., Talantseva O.I., Zhukova M.A., Grigorenko E.L. (2023). Endogenous Oxytocin and Human Social Interactions: A Systematic Review and Meta-Analysis. Psychol. Bull..

[32] Gallagher S., Varga S. (2015). Social Cognition and Psychopathology: A Critical Overview. World Psychiatry.

[33] Haza B., Gosling C.J., Ciminaghi F., Conty L., Pinabiaux C. (2024). Research Review: Social Cognition and Everyday Social Skills in Children and Adolescents with Attention-deficit/Hyperactivity Disorder: A Meta-analysis of Case–Control Studies. Child Psychol. Psychiatry.

[34] Nejati V. (2022). Reading Mind from the Eyes in Individuals with Attention Deficit-Hyperactivity Disorder (ADHD): A Meta-Analysis. Expert Rev. Neurother..

[35] Bora E., Pantelis C. (2016). Meta-Analysis of Social Cognition in Attention-Deficit/Hyperactivity Disorder (ADHD): Comparison with Healthy Controls and Autistic Spectrum Disorder. Psychol. Med..

[36] Casula A., Belluardo G., Antenucci C., Bianca F., Corallo F., Ferraioli F., Gargano D., Giuffrè S., Giunta A.L.C., La Torre A. (2025). The Role of Empathy in ADHD Children: Neuropsychological Assessment and Possible Rehabilitation Suggestions—A Narrative Review. Medicina.

[37] Dessoki H.H., Amin O.R., Soltan M.R., Abbas M.M., Dawoud M.E. (2020). Social Cognitive Deficits in Male Children with Attention Deficit Hyperactivity Disorder in Relation to Salivary Oxytocin Level. Middle East Curr. Psychiatry.

[38] Keech B., Crowe S., Hocking D.R. (2018). Intranasal Oxytocin, Social Cognition and Neurodevelopmental Disorders: A Meta-Analysis. Psychoneuroendocrinology.

[39] Sasaki T., Hashimoto K., Oda Y., Ishima T., Kurata T., Takahashi J., Kamata Y., Kimura H., Niitsu T., Komatsu H. (2015). Decreased Levels of Serum Oxytocin in Pediatric Patients with Attention Deficit/Hyperactivity Disorder. Psychiatry Res..

[40] Wernicke J., Zhang Y., Felten A., Du J., Yao S., Kou J., Chen Y., Kendrick K.M., Becker B., Reuter M. (2020). Blood Oxytocin Levels Are Not Associated with ADHD Tendencies and Emotionality in Healthy Adults. Neurosci. Lett..

[41] Boyle D., Levi-Shachar O., Gvirts H.Z., Zagoory-Sharon O., Feldman R., Bloch Y., Nitzan U., Maoz H. (2021). Lack of Association between Severity of ADHD Symptoms and Salivary Oxytocin Levels. Psychoneuroendocrinology.

[42] Alkalay S., Dan O. (2022). Effect of Short-Term Methylphenidate on Social Impairment in Children with Attention Deficit/Hyperactivity Disorder: Systematic Review. Child. Adolesc. Psychiatry Ment. Health.

[43] Levi-Shachar O., Gvirts H.Z., Goldwin Y., Bloch Y., Shamay-Tsoory S., Zagoory-Sharon O., Feldman R., Maoz H. (2020). The Effect of Methylphenidate on Social Cognition and Oxytocin in Children with Attention Deficit Hyperactivity Disorder. Neuropsychopharmacology.

[44] Demirci E., Ozmen S., Kilic E., Oztop D.B. (2016). The Relationship between Aggression, Empathy Skills and Serum Oxytocin Levels in Male Children and Adolescents with Attention Deficit and Hyperactivity Disorder. Behav. Pharmacol..

[45] Eom T.H., Kim Y.-H. (2024). Clinical Practice Guidelines for Attention-Deficit/Hyperactivity Disorder: Recent Updates. Clin. Exp. Pediatr..

[46] Kaufman J., Townsend L.D., Kobak K. (2017). The Computerized Kiddie Schedule for Affective Disorders and Schizophrenia (KSADS): Development and Administration Guidelines. J. Am. Acad. Child Adolesc. Psychiatry.

[47] Unal F., Oktem F., Cetin Cuhadaroglu F., Cengel Kultur S.E., Akdemir D., Foto Ozdemir D., Cak H.T., Unal D., Tiras K., Aslan C. (2019). Reliability and Validity of the Schedule for Affective Disorders and Schizophrenia for School-Age Children-Present and Lifetime Version, DSM-5 November 2016-Turkish Adaptation (K-SADS-PL-DSM-5-T). Turk. J. Psychiatry.

[48] Akçamete G.A.H. (2016). Sosyal Becerileri Değerlendirme Ölçeği’nin (7–12 Yaş) Geçerlik ve Güvenirlik Çalışması.

[49] Baron-Cohen S., Wheelwright S., Hill J., Raste Y., Plumb I. (2001). The “Reading the Mind in the Eyes” Test Revised Version: A Study with Normal Adults, and Adults with Asperger Syndrome or High-Functioning Autism. J. Child Psychol. Psychiatry.

[50] Girli A. (2014). Psychometric Properties of the Turkish Child and Adult Form of “Reading the Mind in the Eyes Test”. Psych.

[51] Bryant B.K. (1982). An Index of Empathy for Children and Adolescents. Child Dev..

[52] Yüksel A. (2003). Empati Eğitim Programının İlköğretim Öğrencilerinin Empatik Becerilerine Etkisi. Ph.D. Thesis.

[53] Bussing R., Fernandez M., Harwood M., Wei H., Garvan C.W., Eyberg S.M., Swanson J.M. (2008). Parent and Teacher SNAP-IV Ratings of Attention Deficit Hyperactivity Disorder Symptoms: Psychometric Properties and Normative Ratings From a School District Sample. Assessment.

[54] Swanson J.M., Schuck S., Porter M.M., Carlson C., Hartman C.A., Sergeant J.A., Clevenger W., Wasdell M., McCleary R., Lakes K. (2012). Categorical and Dimensional Definitions and Evaluations of Symptoms of ADHD: History of the SNAP and the SWAN Rating Scales. Int. J. Educ. Psychol. Assess..

[55] Tabak B.A., Leng G., Szeto A., Parker K.J., Verbalis J.G., Ziegler T.E., Lee M.R., Neumann I.D., Mendez A.J. (2023). Advances in Human Oxytocin Measurement: Challenges and Proposed Solutions. Mol. Psychiatry.

[56] Horvat-Gordon M., Granger D.A., Schwartz E.B., Nelson V.J., Kivlighan K.T. (2005). Oxytocin Is Not a Valid Biomarker When Measured in Saliva by Immunoassay. Physiol. Behav..

[57] Bowen R.A.R., Remaley A.T. (2014). Interferences from Blood Collection Tube Components on Clinical Chemistry Assays. Biochem. Med..

[58] Arango-Tobón O.E., Guevara Solórzano A., Orejarena Serrano S.J., Olivera-La Rosa A. (2023). Social Cognition and Prosocial Behavior in Children with Attention Deficit Hyperactivity Disorder: A Systematic Review. Healthcare.

[59] Humphreys K.L., Katz S.J., Lee S.S., Hammen C., Brennan P.A., Najman J.M. (2013). The Association of ADHD and Depression: Mediation by Peer Problems and Parent–Child Difficulties in Two Complementary Samples. J. Abnorm. Psychol..

[60] Shang C.-Y., Shih H.-H., Pan Y.-L., Lin H.-Y., Gau S.S.-F. (2020). Comparative Efficacy of Methylphenidate and Atomoxetine on Social Adjustment in Youths with Attention-Deficit/Hyperactivity Disorder. J. Child Adolesc. Psychopharmacol..

[61] Pitzianti M.B., Spiridigliozzi S., Bartolucci E., Esposito S., Pasini A. (2020). New Insights on the Effects of Methylphenidate in Attention Deficit Hyperactivity Disorder. Front. Psychiatry.

[62] Aduen P.A., Day T.N., Kofler M.J., Harmon S.L., Wells E.L., Sarver D.E. (2018). Social Problems in ADHD: Is It a Skills Acquisition or Performance Problem?. J. Psychopathol. Behav. Assess..

[63] Kratochvil C.J., Faries D., Vaughan B., Perwien A., Busner J., Saylor K., Kaplan S., Buermeyer C., Swindle R. (2007). Emotional Expression During Attention-Deficit/Hyperactivity Disorders Treatment: Initial Assessment of Treatment Effects. J. Child Adolesc. Psychopharmacol..

[64] Wehmeier P.M., Schacht A., Lehmann M., Dittmann R.W., Silva S.G., March J.S. (2008). Emotional Well-Being in Children and Adolescents Treated with Atomoxetine for Attention-Deficit/Hyperactivity Disorder: Findings from a Patient, Parent and Physician Perspective Using Items from the Pediatric Adverse Event Rating Scale (PAERS). Child. Adolesc. Psychiatry Ment. Health.

[65] Golubchik P., Weizman A. (2017). The Possible Effect of Methylphenidate Treatment on Empathy in Children Diagnosed with Attention-Deficit/Hyperactivity Disorder, Both With and Without Comorbid Oppositional Defiant Disorder. J. Child Adolesc. Psychopharmacol..

[66] Fantozzi P., Sesso G., Muratori P., Milone A., Masi G. (2021). Biological Bases of Empathy and Social Cognition in Patients with Attention-Deficit/Hyperactivity Disorder: A Focus on Treatment with Psychostimulants. Brain Sci..

[67] Gumustas F., Yilmaz I., Yulaf Y., Gokce S., Sabuncuoglu O. (2017). Empathy and Facial Expression Recognition in Children with and Without Attention-Deficit/Hyperactivity Disorder: Effects of Stimulant Medication on Empathic Skills in Children with Attention-Deficit/Hyperactivity Disorder. J. Child Adolesc. Psychopharmacol..

[68] Belal M., Moussa S., Omnia R.A., Fakher W. (2025). Salivary Oxytocin Levels, Empathy, and Executive Functions in Egyptian Children with ADHD: A Case–Control Study. Middle East Curr. Psychiatry.

